# Supplementary material for: Structural basis of semaphorin‐plexin cis interaction
Source: EMBO J. 2020 Jun 5;39(13):e102926. doi: 10.15252/embj.2019102926 (PMC7327498; doi:10.15252/embj.2019102926)
Supplement: Supplementary file 1 — Expanded View Figures PDF [file EMBJ-39-e102926-s001.pdf]

Expanded View Figures

Figure EV1. Sema1b is a monomer on the cell surface.

A Number and Brightness analysis was used to determine the molecular brightness (ε) of Sema1b-mClover or Sema1b-F254C-mClover in live COS-7 cells. Sema1b seems to be a monomer on the cell surface as the average ε value for Sema1b-mClover (ε_{av} = 0.033) is half of the dimeric Sema1b-F254C-mClover (ε_{av} = 0.061). The box limits indicate the 25th and 75th percentiles, centred lines show the median, squares represent sample means, whiskers extend 1.5-fold the interquartile range from the 25th and 75th percentiles, and *P*-value was calculated by one-way analysis of variance (ANOVA).

B Representative images of COS-7 cells transiently expressing Sema1b-mClover or Sema1b-F254C-mClover in the Number and Brightness experiment. The average intensity images (first column from the left, grey colour) are shown along with the brightness images (second column from the left, rainbow pseudocolor). Scale bar, 40 μm.

C Number and Brightness analysis shows that Sema1b$_{ecto}$ fails to dimerise PlexA-mClover on the cell surface of COS-7 cells contrary to the dimeric Sema1b$_{ecto}$-F254C. The PlexA-mClover construct contained the ectodomain followed by a transmembrane segment and the C-terminal fluorescent protein mClover. The box limits indicate the 25th and 75th percentiles, centred lines show the median, squares represent sample means, whiskers extend 1.5-fold the interquartile range from the 25th and 75th percentiles, and *P*-value was calculated by one-way analysis of variance (ANOVA).

D Dimerisation of PlexA analysed by Number and Brightness assay. Representative images of COS-7 cells transiently expressing PlexA-mClover before and after treatment with monomeric Sema1b$_{ecto}$ or dimeric Sema1b$_{ecto}$-F254C. The average intensity images (first and second column from the left, grey colour) are shown along with the brightness images (third and fourth column from the left, rainbow pseudocolor). Scale bar, 40 μm.

E Size exclusion chromatography with multi-angle light scattering of PlexA$_{1-4}$ showing the molar mass and elution profiles at three initial PlexA$_{1-4}$ concentrations of 2.0 (red), 1.0 (blue) and 0.5 mg/ml (green). Experimental mass of 75 kDa indicates that PlexA$_{1-4}$ is a monomer.

F Sedimentation coefficient distribution of PlexA$_{1-4}$ determined by sedimentation velocity analytical ultracentrifugation at a concentration of 33 μM. Calculated sedimentation coefficient (s_w(20,w) = 5.0 S) corresponds to the predicted sedimentation coefficient for the monomer.

G Zoomed-in view of the side-on interface showing three main binding sites. Interacting residues are shown in purple.

H Superposition showing the side-on Sema1b-PlexA (blue-orange) interaction from the 2:2 complex overlayed onto the *Drosophila* Sema2a dimer (pdb 6qp7) structure (grey). The superposition is based on the Sema1b and Sema2a (chain A) sema domains. The clashes between PlexA and Sema2a (chain B) indicate that the side-on binding mode only becomes possible if a semaphorin is in the monomeric state, because the interaction site is otherwise occluded by the dimer interface.

 © 2020 The Authors

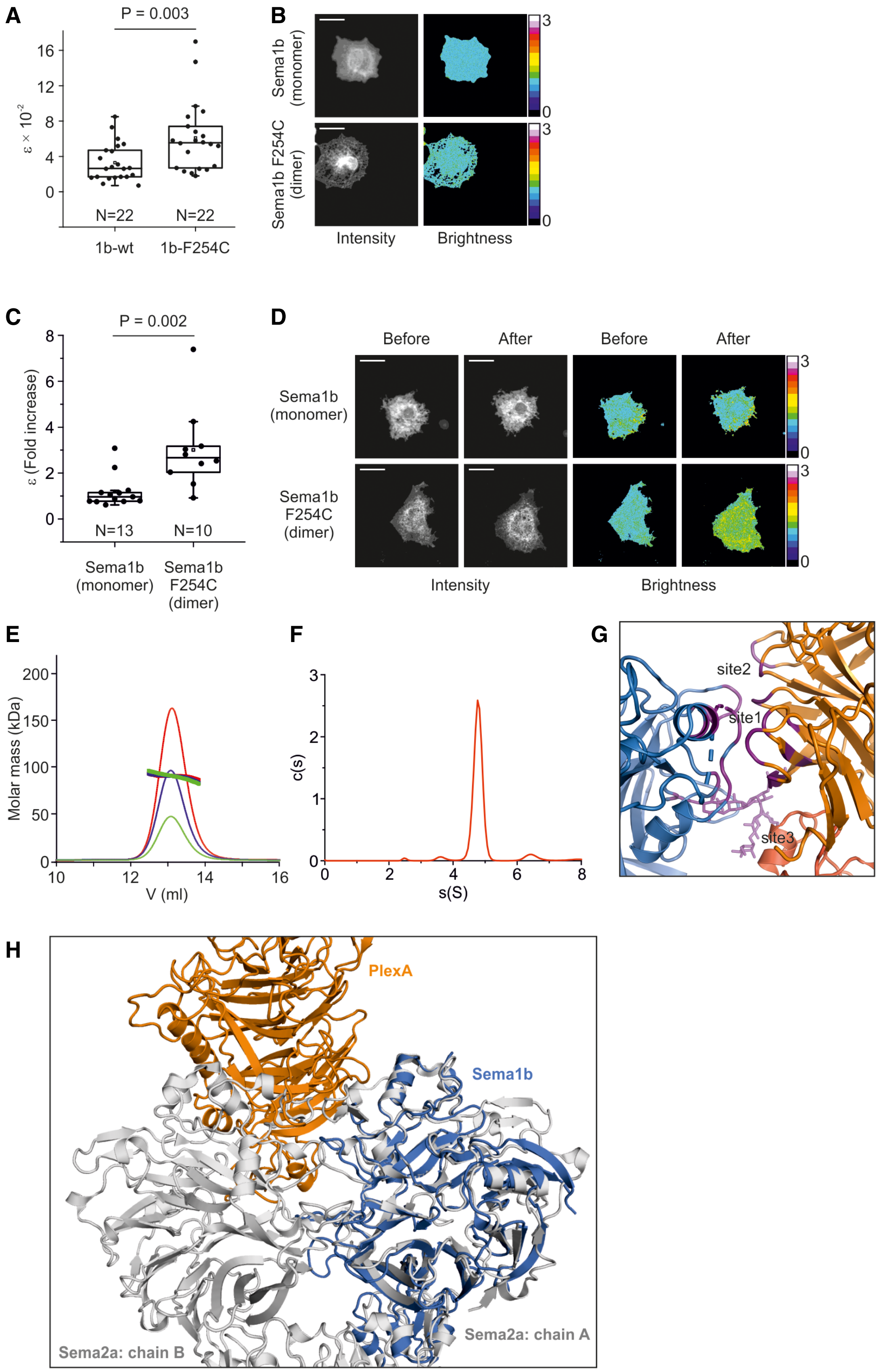
A
P = 0.003
ε × 10⁻²
16
12
8
4
0
N=22
N=22
1b-wt
1b-F254C
B
Sema1b (monomer)
Sema1b F254C (dimer)
Intensity
Brightness
3
0
C
P = 0.002
ε (Fold increase)
8
6
4
2
0
N=13
N=10
Sema1b (monomer)
Sema1b F254C (dimer)
D
Before
After
Before
After
Sema1b (monomer)
Sema1b F254C (dimer)
Intensity
Brightness
E
Molar mass (kDa)
200
150
100
50
0
10
12
14
16
V (ml)
F
c(s)
3
2
1
0
0
2
4
6
8
s(S)
G
site2
site1
site3
H
PlexA
Sema1b
Sema2a: chain B
Sema2a: chain A

Figure EV1.

Figure EV2. Sema1b binds PlexA at two independent sites.

A Ribbon representation of the PlexA-Sema1b side-on orientation.

B The positioning of Nrp1, which is wedged between the sema domains of semaphorin and plexin, in the Sema3A-PlxnA2-Nrp1 ternary complex (pdb 4gza) in ribbon representation.

C Superposition of (A) and (B) on the basis of the sema domains of Sema1b and Sema3A. Superposition shows that the neuropilin binding site and the interaction site B in the side-on orientation are in very close proximity without any significant steric clashes.

D–L Microscale thermophoresis binding experiment for $PlexA_{1\text{-}4}$-mVenus and $Sema1b_{ecto}$-mutA (D-F) or $Sema1b_{ecto}$-mutB (G-I) or $Sema1b_{ecto}$-mutA+B (J-L). (D, G, J) Capillary scans of $PlexA_{1\text{-}4}$-mVenus in standard capillaries. (E, H, K) Fluorescence time traces recorded by MST instrument. The cold and hot regions used for fitting are shown by blue and red vertical lines, respectively. Close-up views of the hot regions are shown in the middle. (F, I, L) The binding curves of $Sema1b_{ecto}$ to $PlexA_{1\text{-}4}$-mVenus. Error bars represent s.d. of three technical replicates.

M–O Size exclusion chromatography with multi-angle light scattering of $Sema1b_{1\text{-}2}$ (M), $PlexA_{1\text{-}4}$ (N), and an equimolar mixture of $Sema1b_{1\text{-}2}$ and $PlexA_{1\text{-}4}$ (O). Panels (M) and (N) show the molar mass and elution profiles at three initial concentrations of 2.0 (red), 1.0 (blue) and 0.5 mg/ml (green). Experimental mass of 75 kDa for $PlexA_{1\text{-}4}$ and experimental mass of 60 kDa for $Sema1b_{1\text{-}2}$ indicate that both $PlexA_{1\text{-}4}$ and $Sema1b_{1\text{-}2}$ are monomers in solution. The sample containing the equimolar mixture of $Sema1b_{1\text{-}2}$ and $PlexA_{1\text{-}4}$ (O) revealed three peaks corresponding to unliganded $Sema1b_{1\text{-}2}$ and $PlexA_{1\text{-}4}$ and $Sema1b_{1\text{-}2}$ in complex with $PlexA_{1\text{-}4}$ with experimental mass of 131 kDa corresponding to 1:1 stoichiometry.

 © 2020 The Authors

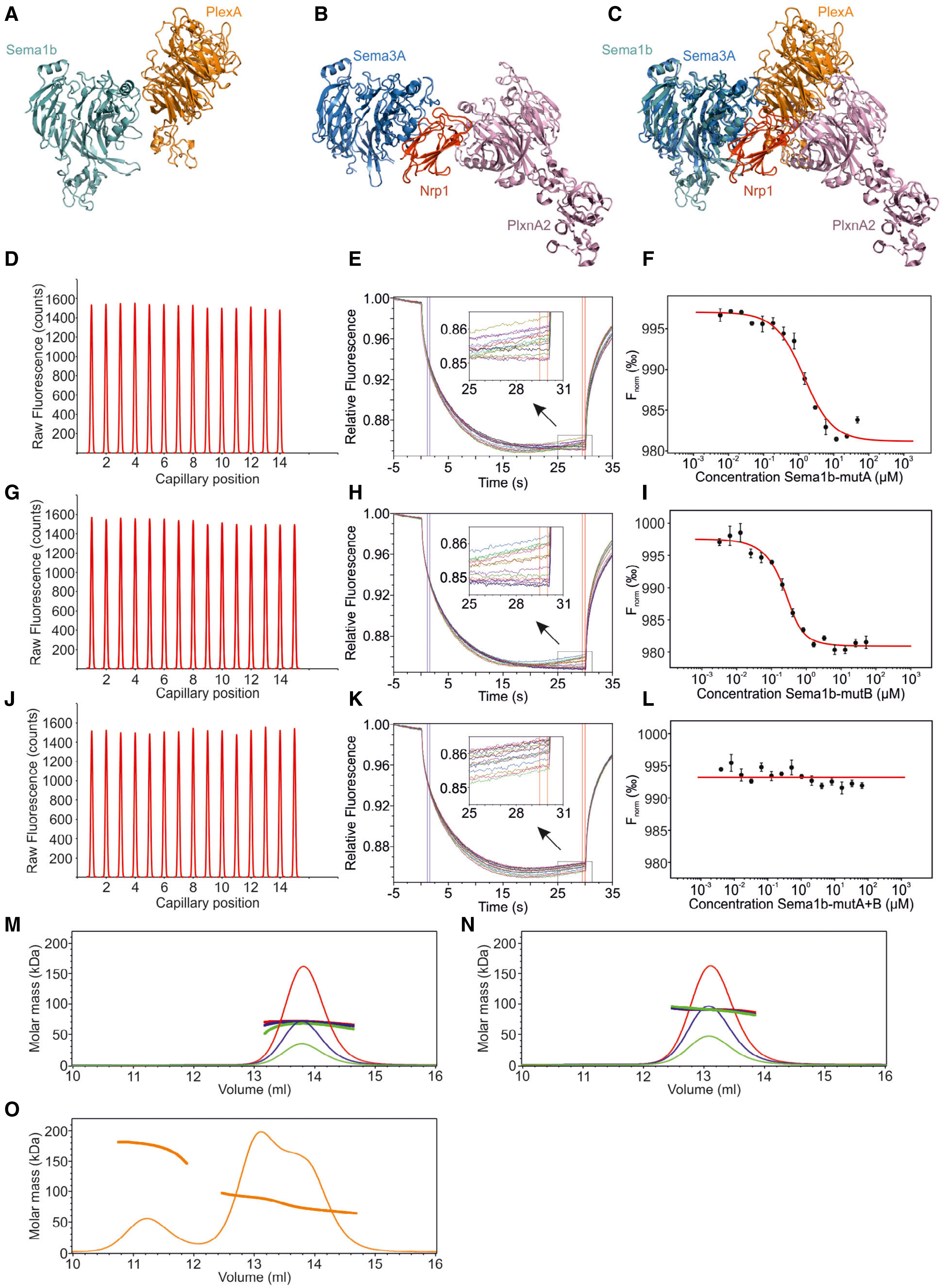
A
B
C
Sema1b
PlexA
Sema3A
Nrp1
PlxnA2
D
E
F
G
H
I
J
K
L
M
N
O
Raw Fluorescence (counts)
Capillary position
Relative Fluorescence
Time (s)
Concentration Sema1b-mutA (μM)
Concentration Sema1b-mutB (μM)
Concentration Sema1b-mutA+B (μM)
Molar mass (kDa)
Volume (ml)

Figure EV2.

Figure EV3. Binding of Sema1b and PlexA in *cis* leads to opening of the PlexA ring-like conformation.

A Representative intensity and FLIM images of COS-7 cells transiently expressing FRET donor (PlexA-mClover), FRET acceptor (Sema1b-mRuby2 or Sema1a-mRuby2) or tandem mClover-mRuby2.The FLIM images are pseudocolored. Scale bar, 20 µm.

B Calculated lifetimes are independent of donor/acceptor fluorescence intensity ratio as shown by the random distribution of the PlexA-mClover/Sema1b-mRuby2 intensity ratio against the average lifetime. The scatter chart was made from 23 randomly sampled cells.

C Distribution of the average fraction of the interacting donor (f_D) in the FRET-FLIM experiment. The boxcharts represent the average lifetime. Box limits indicate the 25th and 75th percentiles, centred lines show the median, squares represent sample means, whiskers extend 1.5-fold the interquartile range from the 25th and 75th percentiles, and *P*-value calculated by one-way analysis of variance (ANOVA). ***$P < 0.001$.

D Representative average fluorescence intensity images showing expression of Sema1b-wild-type-mClover, Sema1b-mutA-mClover, Sema1b-mutB-mClover or Sema1b-mutA+B-mClover on the cell surface of live COS7 cells. Scale bar, 10 µm.

E Distribution of the average fluorescence intensity of Sema1b and all three mutants on the cell surface of live COS-7 cells. The images were collected from two independent experiments. The box limits indicate the 25th and 75th percentiles, centred lines show the median, squares represent sample means, whiskers extend 1.5-fold the interquartile range from the 25th and 75th percentiles, and *P*-value was calculated by *t*-test, ns: $P > 0.05$

F Structural superposition of Sema1b, Sema3A (4gz8), Sema6A (3okw), Sema4D (1olz) and Sema7A (3nvq) indicating the αEx1-αEx2 loop in Sema1b or corresponding loop in the other semaphorins. The loop is involved in the semaphorin homodimerisation in Sema3A, Sema4D and Sema6A but not in Sema7A and A39R (not shown). However, in monomeric Sema1b, the loop is accessible for engagement with PlexA through the interaction site B.

G Representative negative stain electron micrograph of $PlexA_{ecto}$ particles. Scale bar, 100 nm.

H Negative stain 2D class averages of $PlexA_{ecto}$ obtained by classifying 12,164 particles into 50 classes. The number of particles within each class is listed in the bottom right corner. Scale bar, 10 nm.

I Sedimentation coefficient distribution of $PlexA_{ecto}$ determined by sedimentation velocity analytical ultracentrifugation at a concentration of 2.2 µM. The predicted sedimentation coefficients of different oligomeric states that best correspond to the peak values are indicated by asterisk.

J–N COS-7 cells collapse assay showing representative images of collapsed and non-collapsed cells (second and third column from the left, pseudocolor) and average intensity images of non-collapsed cells (first column from the left, grey colour). COS-7 cells transiently expressing $PlexA_{FL}$-mClover (J) or co-expressing $PlexA_{FL}$-mClover with Sema1b wild type (K) or Sema1b mutants (L,M,N) were treated with purified Sema1a-Fc at a final concentration of 5.8 µM. Images were acquired every minute for 30 min. Cell surface area was calculated using ImageJ before and after stimulation. Scale bar, 30 µm.

O Relative change of COS-7 cells surface area upon treatment with vehicle only. Cells expressing $PlexA_{FL}$-mClover or $PlexA_{FL}$-mCover and Sema1b-mRuby2 wild-type or mutants were treated with vehicle only (medium without semaphorin). Images were acquired every minute for 30 min. Cell surface area was calculated using ImageJ before and after stimulation. Data are presented as means ± SEM. The images were collected from two independent experiments.

 © 2020 The Authors

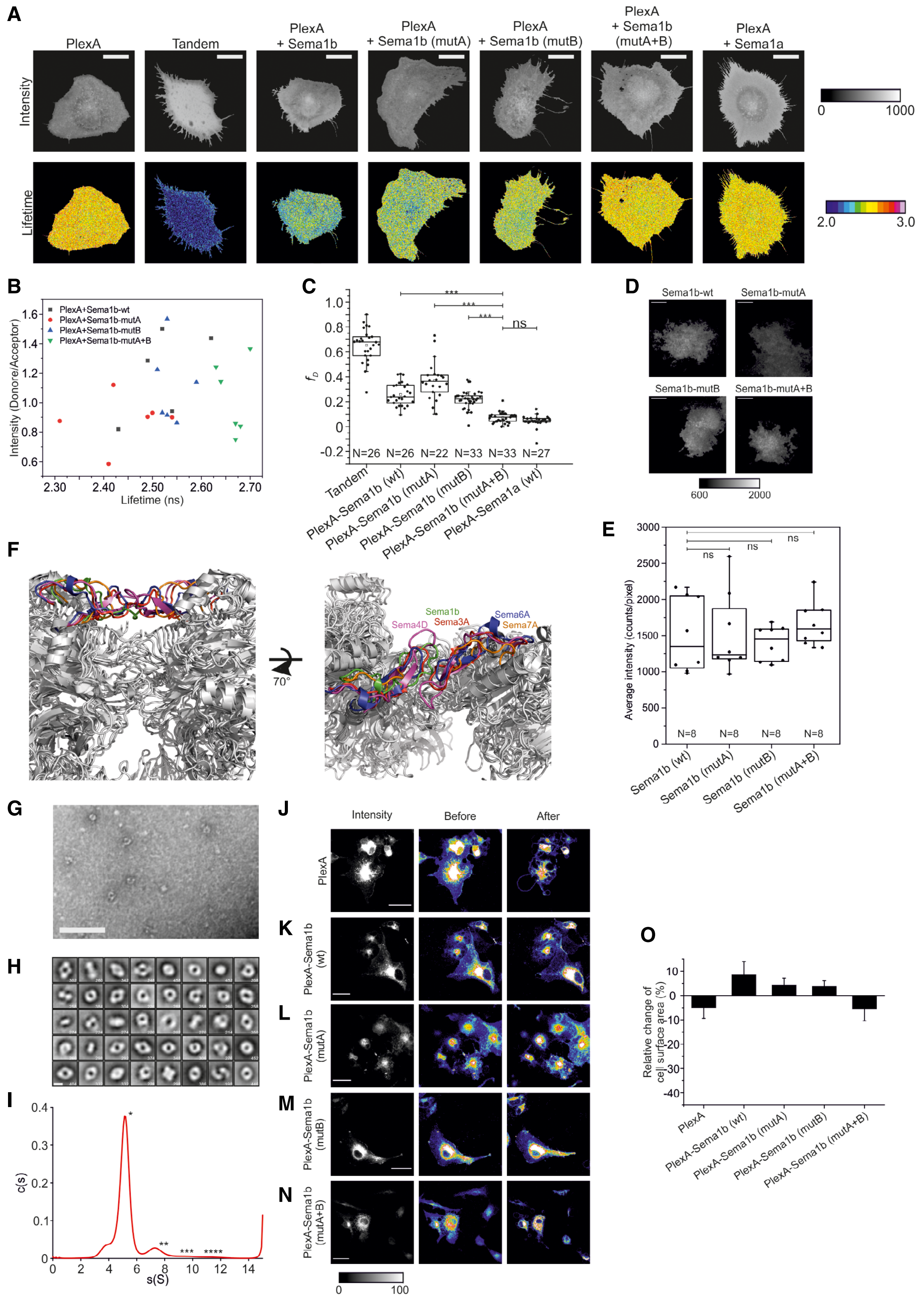
A
PlexA
Tandem
PlexA + Sema1b
PlexA + Sema1b (mutA)
PlexA + Sema1b (mutB)
PlexA + Sema1b (mutA+B)
PlexA + Sema1a
Intensity
Lifetime
0
1000
2.0
3.0
B
PlexA+Sema1b-wt
PlexA+Sema1b-mutA
PlexA+Sema1b-mutB
PlexA+Sema1b-mutA+B
Intensity (Donore/Acceptor)
Lifetime (ns)
C
***
ns
N=26 N=26 N=22 N=33 N=33 N=27
Tandem
PlexA-Sema1b (wt)
PlexA-Sema1b (mutA)
PlexA-Sema1b (mutB)
PlexA-Sema1b (mutA+B)
PlexA-Sema1a (wt)
D
Sema1b-wt
Sema1b-mutA
Sema1b-mutB
Sema1b-mutA+B
600
2000
E
Average intensity (counts/pixel)
N=8
Sema1b (wt)
Sema1b (mutA)
Sema1b (mutB)
Sema1b (mutA+B)
F
Sema1b
Sema4D
Sema3A
Sema6A
Sema7A
70°
G
H
I
c(s)
s(S)
J
Intensity
Before
After
PlexA
K
PlexA-Sema1b (wt)
L
PlexA-Sema1b (mutA)
M
PlexA-Sema1b (mutB)
N
PlexA-Sema1b (mutA+B)
0
100
O
Relative change of cell surface area (%)
PlexA
PlexA-Sema1b (wt)
PlexA-Sema1b (mutA)
PlexA-Sema1b (mutB)
PlexA-Sema1b (mutA+B)

Figure EV3.

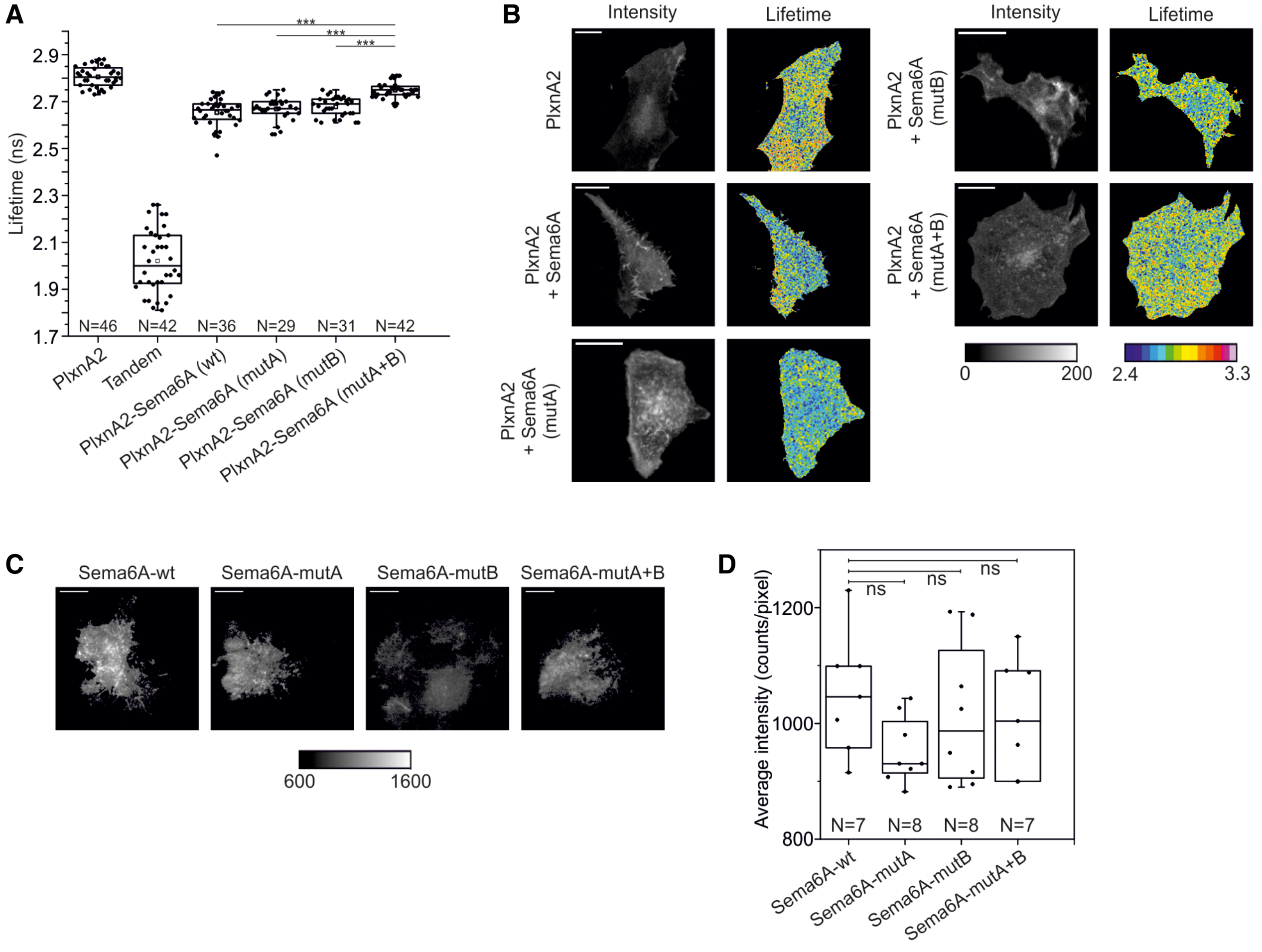

Figure EV4. Mouse Sema6A utilises the same head-to-head and side-on binding modes to interact with PlxnA2 in *cis* as that observed for *Drosophila* Sema1b.

A FRET-FLIM in live COS-7 cells shows cell surface Sema6A-PlxnA2 *cis* interaction. Sema6A mutants reveal that both head-to-head and side-on orientations are involved in the *cis* interaction. The boxcharts represent the average lifetime. Box limits indicate the 25th and 75th percentiles, centred lines show the median, squares represent sample means, whiskers extend 1.5-fold the interquartile range from the 25th and 75th percentiles. ***$P < 0.001$, P-value calculated by one-way analysis of variance (ANOVA).

B Representative time correlated single photon counting intensity (first column from the left) and FLIM images (second column from the left) of COS-7 cells transiently expressing FRET donor (PlxnA2-mClover), FRET acceptor (Sema1b-mRuby2 or Sema1a-mRuby2) or tandem mClover-mRuby2. The FLIM images are pseudocolored. Scale bar, 20 μm.

C Representative average fluorescence intensity images showing co-expression of PlxnA2 with Sema6A-wild-type-mClover, Sema6A-mutA-mClover, Sema6A-mutB-mClover or Sema6A-mutA+B-mClover on the cell surface of live COS7 cells. Scale bar, 10 μm.

D Distribution of the average fluorescence intensity of Sema6A and all three mutants on the cell surface of live COS-7 cells. The images were collected from two independent experiments. The box limits indicate the 25th and 75th percentiles, centred lines show the median, squares represent sample means, whiskers extend 1.5-fold the interquartile range from the 25th and 75th percentiles, and P-value was calculated by t-test, ns: $P > 0.05$.

 © 2020 The Authors

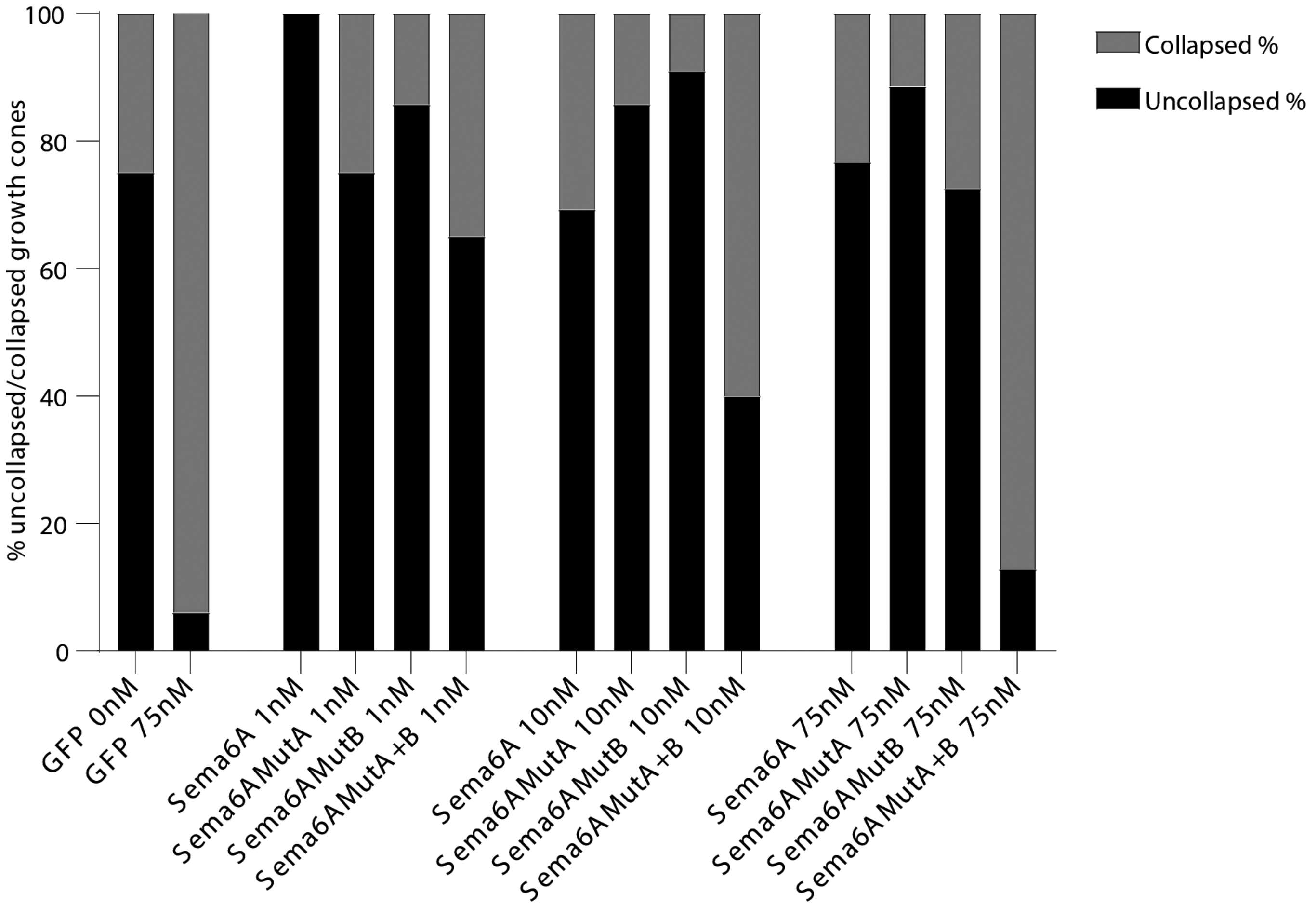

Figure EV5. DRG neurons transfected with Sema6A-mutA+B show growth cone collapse with increasing Sema6A-Fc concentrations.

Growth cone collapse responses for DRG neurons from E12.5 *Sema6A* KO embryos transfected with EGFP-pCAG (GFP), Sema6A-WT-EGFP-pCAG (Sema6A), Sema6A-mutA-EGFP-pCAG (Sema6AMutA), Sema6A-mutB-EGFP-pCAG (Sema6AMutB) or Sema6A-mutA+B-EGFP-pCAG (Sema6AMutA+B) constructs followed by stimulation with different Sema6A-Fc protein concentrations (1, 10 or 75 nM) (see also Fig 3 for a more extensive analysis at 75 nM). DRG neurons transfected with EGFP-pCAG displayed robust collapse following treatment with 75 nM Sema6A-Fc, as compared to control (0 nM). Transfection of Sema6A-WT-EGFP-pCAG, Sema6A-mutA-EGFP-pCAG, or Sema6A-mutB-EGFP-pCAG inhibited Sema6A-Fc-induced growth cone collapse. In contrast, DRG neurons transfected with Sema6A-mutA+B-EGFP-pCAG acquired collapse sensitivity with increasing ligand concentrations. Growth cone collapse was assessed using the growth cone matrix shown in Fig 3 (category 1–4; uncollapsed, category 5–8; collapsed). 4–10 growth cones were analysed per condition per experiment in 2–3 independent experiments.

© 2020 The Authors
